# Event-Based Control Strategy for Mobile Robots in Wireless Environments

**DOI:** 10.3390/s151229796

**Published:** 2015-12-02

**Authors:** Rafael Socas, Sebastián Dormido, Raquel Dormido, Ernesto Fabregas

**Affiliations:** Departamento de Informática y Automática, Universidad Nacional de Educación a Distancia, Juan del Rosal 16, Madrid 28040, Spain; sdormido@dia.uned.es (S.D.); raquel@dia.uned.es (R.D.); efabregas@bec.uned.es (E.F.)

**Keywords:** event-based control, navigation algorithms, mobile robots, wireless communication

## Abstract

In this paper, a new event-based control strategy for mobile robots is presented. It has been designed to work in wireless environments where a centralized controller has to interchange information with the robots over an RF (radio frequency) interface. The event-based architectures have been developed for differential wheeled robots, although they can be applied to other kinds of robots in a simple way. The solution has been checked over classical navigation algorithms, like wall following and obstacle avoidance, using scenarios with a unique or multiple robots. A comparison between the proposed architectures and the classical discrete-time strategy is also carried out. The experimental results shows that the proposed solution has a higher efficiency in communication resource usage than the classical discrete-time strategy with the same accuracy.

## 1. Introduction

A distributed networked control system (NCS) is composed of numerous subsystems (fixed or mobile) called agents, which are geographically distributed. In such a system, the individual subsystems exchange information over a communication network. The communication network generally uses wireless technology, which has a limited bandwidth and constraints in throughput and delays. In practice, communication, especially in wireless networks, uses digital network infrastructures, where the information is transmitted in discrete packets. These packets may be lost during communication. Moreover, the communication media are a resource that is usually accessed in an exclusive manner by the agents. This means that the throughput capacity of such networks and the number of elements is limited. In this kind of network, there are effects of non-linearities and constraints in closed loop control that may impact the performance and control stability of the system. Some effects are the loss of information when transmitting data, variable communication delays, signal sampling and quantization issues due to information packaging and constraints related to limited bandwidth [1]. On the other hand, it is important to decrease the input traffic to the network in a short period of time. This means that the input data to a network will be reduced. An important result of this kind of reduction is that the network can simply guarantee a predictable bandwidth to a control loop, and furthermore, it simplifies the analysis of network delay, which affects the control loop [2]. For these reasons, an important issue in the implementation of these systems is to define methods that use the limited network resources available for transmitting the state and control information more effectively.

To deal with this problem, the timing issue in NCS has been investigated by some researches [3]. In traditional approaches, the controllers are used under the assumption of perfect communication, and then, the maximum allowable transfer interval (MATI) between two subsequent message transmissions that ensures closed loop stability under a network protocol is determined. Examples of them are the try once discard (TOD) or round robin (RR) protocol. The MATI scheme is often done in a centralized manner; therefore, it is impractical for large-scale systems. Moreover, because the MATI is computed before the system is deployed, it must ensure performance levels over all possible system states. As a result, the MATI may be conservative in the sense of being shorter than necessary to assure a specified performance level. Consequently, the bandwidth of the network has to be higher than necessary to ensure the MATI is not violated.

Many others authors have achieved an important reduction of bandwidth utilization without significant loss of performance. Two approaches have been considered: model-based networked control systems (MBNCS) and event-based control. The MBNCS approach was introduced in [4,5], and it was considered in networks of coupled systems [6] using periodic communication. Another philosophy to deal this problem is based on an event-based feedback scheme in NCS [7,8,9,10]. In event-based systems, an agent broadcasts its state information only when it is needed. In this case, this means that some measure of the agent’s state error is above a specified threshold (event threshold). The architecture is decentralized in the sense that an agent is able to produce broadcast state information using its locally-sampled data. Moreover, the selection of the event threshold only requires local information from the sensors of the agent, so that the design is decentralized.

Event-based control has been widely used for the stabilization of dynamical systems while reducing the number of measurements that the sensors need to send to the controller. The events based on state errors have been used extensively [11]. In [12,13,14], the same ideas have been extended to consider networked interconnected systems. A common characteristic in these works on event-based control is the use of a zero order hold (ZOH) model in the controller node and the assumption that the models being used are the same as the plants that they represent. In recent years, the research interest in the field of event-based control has been growing, and the volume of publications is increasing.

This work presents a new control strategy for mobile robots in wireless environments. The main idea is to develop architectures that manage the limited radio resources efficiently with a similar accuracy as the classical solutions. The event-based control strategies have been investigated to implement navigation algorithms and to compare them to the discrete-time implementations.

The paper is organized as follow. Section 2 shows an overview of the event-based control. In Section 3, the classical control strategies for wireless environments are described. The proposed control strategy is presented in Section 4. In Section 5, the navigation algorithms investigated in this work are presented. The experimental results are discussed in Section 6. Finally, the conclusions and future work are presented in Section 7.

## 2. Event-Based Control Overview

In contrast to the continuous-time or discrete-time control strategies, in event-based control systems [15], information exchanges among the sensors, the controller and the actuators are triggered with dependence on the system behavior, e.g., when the system variables exceed certain tolerance bounds. In other words, the activity of the controller and the communication between the components in the control system are restricted to time intervals in which the controller inevitably must act in a closed loop manner in order to guarantee the desired specifications of the closed loop system.

**Figure 1:**
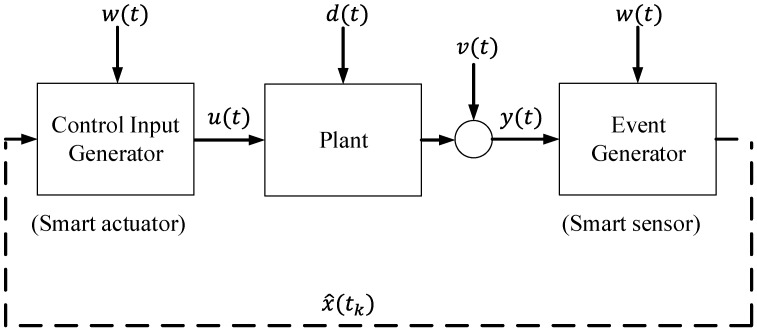
General scheme of an event-based control strategy.

The basic structure of the event-based control strategy is depicted in Figure 1. It consists of the following elements: (1) the plant, (2) the event generator and (3) the control input generator [16]. The plant has input vector u(t), output vector y(t), state vector x(t), disturbance vector d(t) and noise in the sensors v(t). The event generator determines the event times $t_{k}$ at which information $\hat{x}\left(t_{k}\right)$ (estimated state vector) is sent towards the control input generator. The event generator block calculates the difference between the output of the system y(t) and the reference signal w(t). If this value crosses a certain value defined as the event threshold, an event is generated, and the estimated state vector $\hat{x}\left(t_{k}\right)$ is sent to the controller. The control input generator computes the continuous time input u(t) of the plant based on the information obtained at time $t_{k}$ and the command input w(t). See [17] for further information.

In recent years, event-based control has motivated the interest of researchers, and multiple control schemes and new applications have been developed based on these ideas. In [18], an event-driven sampling method called the area-triggered method has been proposed. In this scheme, sensor data are sent only when the integral of differences between the current sensor value and the last transmitted one is greater than a given threshold. The proposed method not only reduces the data transmission rate, but also improves estimation performance in comparison with the conventional event-driven method. The work presented in [19] describes how greenhouse climate control can be represented as an event-based system in combination with wireless sensor networks, where low-frequency dynamics variables have to be controlled, and control actions are mainly calculated against events produced by external disturbances. The proposed control system allows saving costs related to wear minimization and prolonging the actuator life, but keeping promising performance results. Event-based sampling according to a constant energy of sampling error is analyzed in [20]. The defined criterion is suitable for applications where the energy of the sampling error should be bounded (e.g., in building automation or in greenhouse climate monitoring and control). The proposed sampling principle extends a range of event-based sampling schemes and makes the choice of a particular sampling criterion more flexible for the application requirements. Finally, a modified fault isolation filter for a discrete-time networked control system with multiple faults is implemented by a particular form of the Kalman filter in [21]. The proposed fault isolation filter improves the resource utilization with graceful fault estimation performance degradation.

## 3. Control Strategies in Wireless Environments for Mobile Robots

**Figure 2:**
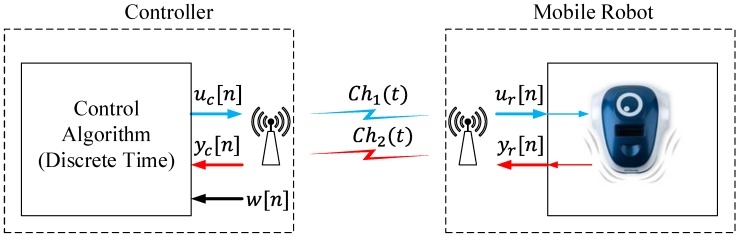
Wireless discrete-time control.

In a wireless environment, the typical architecture for mobile robots is a discrete-time control system, as is shown in Figure 2.

The elements of this system work in the discrete-time domain; the control signals ($u_{c}\left[n\right]$ and $u_{r}\left[n\right]$) and the sensor signals ($y_{r}\left[n\right]$ and $y_{c}\left[n\right]$) have a sampling period of $1/f_{s}$, where $f_{s}$ is the sampling frequency of the system. The RF channels ($Ch_{1}\left(t\right)$ and $Ch_{2}\left(t\right)$) could use analog or digital modulations to transport the information between the elements. In both cases, this RF interface works in the continuous-time domain.

In this scheme, the controller sends the control signals $u_{c}\left[n\right]$ over a communication channel $Ch_{1}\left(t\right)$; this information is received in the robot $u_{r}\left[n\right]$ and acts over the actuators. The difference between $u_{c}\left[n\right]$ and $u_{r}\left[n\right]$ is the noise and the perturbations in the communication channel $Ch_{1}\left(t\right)$. The sensor signals $y_{r}\left[n\right]$ are sent towards the controller over another communication channel $Ch_{2}\left(t\right)$. Finally, the controller receives the sensor signals $y_{c}\left[n\right]$ and calculates the control signals $u_{c}\left[n\right]$, computing the reference signal w[n] and the information from the sensors. As in channel $Ch_{1}\left(t\right)$, the noise and the perturbations in channel $Ch_{2}\left(t\right)$ produce two different signals $y_{r}\left[n\right]$ and $y_{c}\left[n\right]$. This system interchanges information between the controller and the mobile robot every $1/f_{s}$ seconds.

In this structure, the communication channels $Ch_{1}\left(t\right)$ and $Ch_{2}\left(t\right)$ are busy every $1/f_{s}$, because the system is periodically interchanging information between the controller and the robot. When the robot is in a steady state, it is not necessary to interchange information between the robot and the controller, but the channel is busy due to the system sending information over the channels each period of time $1/f_{s}$ unnecessarily. When a wireless infrastructure is used to connect the elements of the control system, it is mandatory to use the radio resources only for critical purposes due to these communication systems frequently having a limited spectrum, and only a few elements can send information at the same time. For this reason, in this work, other control methodologies has been investigated to improve the efficiency in the wireless communication channels.

## 4. Proposed Event-Based Control Strategy in a Wireless Environment

As was mentioned before, if the system is in a steady state, the event-based strategies have two main advantages *versus* the discrete-time control ones: (1) the communication resources are not used and (2) the controller does not need to compute new control signals. For these reasons, in this paper, a new event-based control strategy for mobile robots in wireless environments is proposed.

### 4.1. Control Architectures

**Figure 3:**
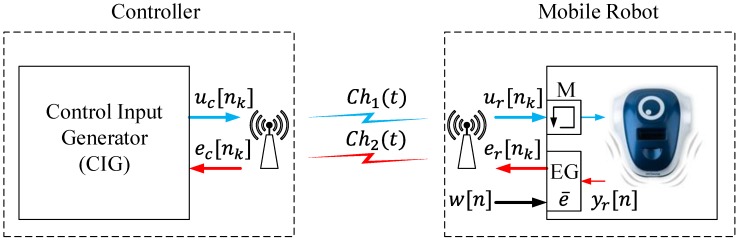
Proposed event-based control strategy.

Based on [22], a new approach for a wireless control system for mobile robots has been proposed. The architecture of the system is depicted in Figure 3.

In the robot, an event generator (EG) compares the sensor signals $y_{r}\left[n\right]$ to the reference signal w[n]. If the difference between both signals crosses the event threshold $\bar{e}$, an event *k* is generated, and the error signal $e_{r}\left[n_{k}\right]$ is sent to the controller over the radio interface. Different methodologies can be used to generate events. In [23], a review of these procedures has been analyzed. In a general way, the event threshold usually is a constant value, and it has to be set carefully. Otherwise, it can be defined as a function of the noise or as a function of other variables to get more accuracy in event generation; see [24,25] for details. The error signal $e_{r}\left[n_{k}\right]$ goes to the controller via the communication channel $Ch_{2}\left(t\right)$. In this case, the channel is busy only when the EG generates events. When the robot is in a steady state, the signals w[n] and $y_{r}\left[n\right]$ are very close, and no event is generated. In this case, the communication channel $Ch_{2}\left(t\right)$ is free. Every time an event is generated and the error signal $e_{r}\left[n_{k}\right]$ is sent to the controller, the robot receives the control signal $u_{r}\left[n_{k}\right]$; this signal is saved in a memory *M*. When an event *k* is generated, the memory is updated with new values, but in the period between events, the saved value in the memory is used to act on the robot.

When an event is generated, the control input generator (CIG) receives the error signal $e_{c}\left[n_{k}\right]$, and the control signal $u_{c}\left[n_{k}\right]$ is calculated; then, this information is sent to the robot via the communication channel $Ch_{1}\left(t\right)$. The communication channel $Ch_{1}\left(t\right)$ and the channel $Ch_{2}\left(t\right)$ are busy only when the events are generated, in other periods of time they are free. As in the discrete-time architecture; the differences between the error signals ($e_{r}\left[n_{k}\right]$, $e_{c}\left[n_{k}\right]$) and the control signals ($u_{c}\left[n_{k}\right]$, $u_{r}\left[n_{k}\right]$) are the noise and the perturbations in the communication channels $Ch_{1}\left(t\right)$ and $Ch_{2}\left(t\right)$.

If the proposed architecture is compared to the discrete-time strategy, it has two main advantages: (1) the communication channels are busy only when the events are generated and (2) the controller does not have to compute control signals when the robot is in the steady state. In this event-based architecture, the reference signal w[n] is computed in the event generator (EG); for this reason, the communication channel $Ch_{1}\left(t\right)$ is used to send this information. In a general way, this information tends to be constant or it changes with a low frequency. In this way, the effects in the usage of the radio resources should be negligible.

The classical control algorithms can be implemented in this scheme in a simple way. Depending on the kind of algorithm to be implemented, the EG and the CIG will be defined in a different way, as is described in Section 5.

### 4.2. Mobile Robot Model

**Figure 4:**
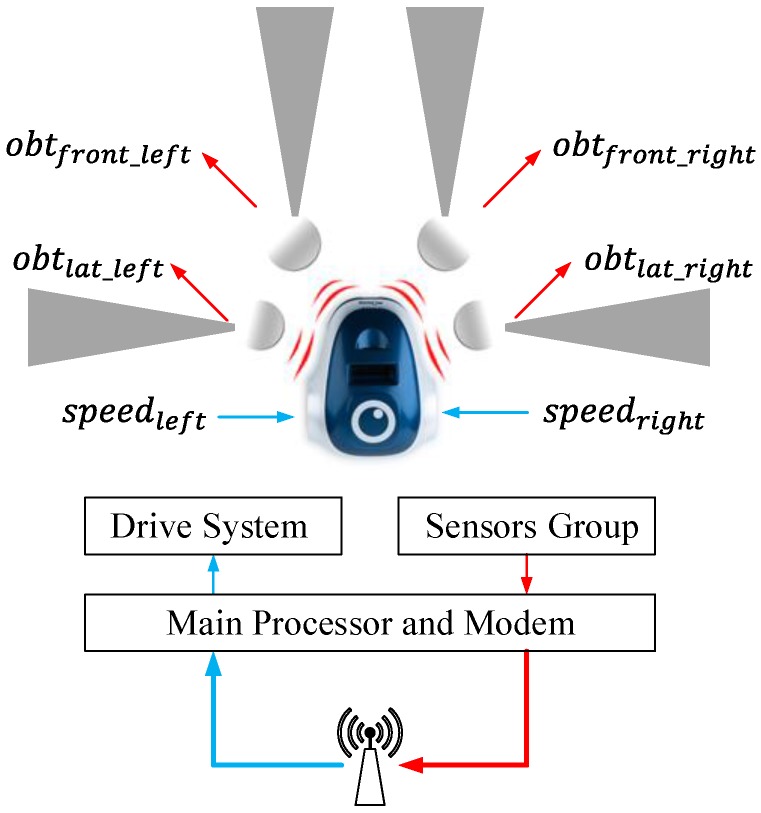
General structure of the differential wheeled robots.

In this work, to check the proposed strategy, some navigation algorithms have been studied. The navigation algorithm will be defined for differential wheeled robots, although the architecture proposed can be adapted to another kind of robot in a simple way. The mobile robot that has been analyzed in this paper has the blocks presented in Figure 4. These robots have a dynamic model, which depends on the length between wheels *L*, the radio *R* and the angular speed of each wheel $speed_{left}$ and $speed_{right}$. In this model, the position in 2D (*x* and *y*) and the heading angle *φ* of the robot can be expressed by Equations (1)–(3).

(1) $$\dot{x}=\frac{R}{2}\left(speed_{right}+speed_{left}\right)\;cos\varphi$$

(2) $$\dot{y}=\frac{R}{2}\left(speed_{right}+speed_{left}\right)\;sin\varphi$$

(3) $$\dot{\varphi }=\frac{R}{L}\left(speed_{right}-speed_{left}\right)$$

The position and orientation of the robot can be managed acting over its angular speeds $speed_{left}$ and $speed_{right}$. In the proposed system, these speeds will be calculated by the controller.

Other important blocks in this robot are the obstacle detection sensors, which use infra-red signals to detect the objects located in front of the robot. Generally, these vehicles have four of these sensors, two on the front $obt_{front\_left}$ and $obt_{front\_right}$ and two on the sides $obt_{lat\_left}$ and $obt_{lat\_right}$. The value obtained from these sensors depends on the proximity of the obstacles: if the distance to the obstacle descends, the value presented by the sensor increases. Other important information that is obtained from these devices is the position of the detected objects, which can be calculated combining the data of the different sensors.

## 5. Navigation Algorithms

In this section, some navigation algorithms will be defined to analyze the proposed architectures. In mobile robot navigation tasks, some well-known navigation algorithms are widely used in this kind of application. Go to goal, wall following and obstacle avoidance are the most commonly used in mobile robot environments [26,27,28]. In this work, some implementations of these algorithms are proposed using the presented event-based architecture. Moreover, a comparison with a discrete-time solution to solve the same problem is presented.

**Figure 5:**
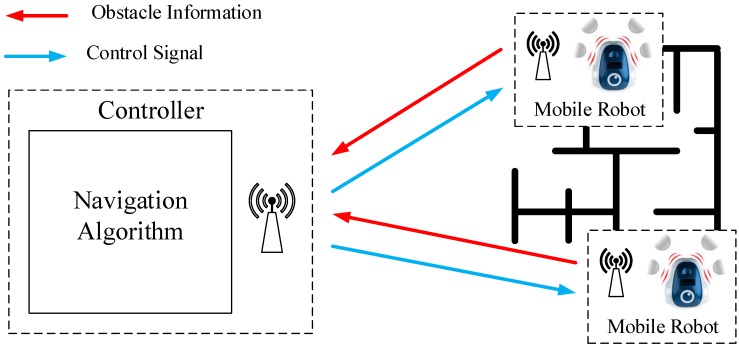
Navigation algorithm implementation based on centralized control.

The architecture used to check these algorithms is depicted in Figure 5. The system consists of a centralized controller and some mobile robots, which use an RF communication between them. The navigation algorithm is implemented in the controller. It sends the control signals (angular speeds) to the robots, and the mobile robots send to the controller the sensor signals (obstacle information). The communication between elements (robots and controller) occurs only when the robot sends sensor information to the controller. When this happens, the controller calculates the control signals and sends this information to the robot. In the discrete-time architecture, the communication between elements is periodical. In the event-based solution proposed in this work, the communication occurs only when the event condition is satisfied.

In the next sections, a wall following and an obstacle avoidance algorithms will be developed to analyze the behavior of the proposed strategy and to compare to a classical discrete-time solution.

### 5.1. Wall Following Algorithm

A wall following algorithm is one of the most common process used in mobile robot navigation. The basic algorithm is as follows:

(1) Find the closest wall.
(2) Move to a desired distance from the wall.
(3) Turn and start moving along the wall, staying the desired distance away.

Wall following algorithms have two variants of implementation: clockwise or counter-clockwise; in this case, the second option is selected. To implement this algorithm in the controller, a simple control law has been designed; it is presented in Equation (4).

(4) $$\begin{array}{c} \mathrm{if}\;(obt_{front\_left}>0)\\ \left\{front\_obstacle=1\right\}\\ \mathrm{else}\;\{front\_obstacle=0\}\\ sc_{left}\left(\%\right)=\left(\frac{obt_{lat\_left}-50}{40}\right)\cdot 40+50\\ sc_{right}\left(\%\right)=50-\left(front\_obstacle\right)\cdot 50 \end{array}$$

In this expression, $obt_{front\_left}$ and $obt_{lat\_left}$ are the values of the sensors, the range of which goes from 0% (obstacle is out of the range of the sensor) to 100% (the obstacle is touching the robot). The auxiliary variable front_obstacle is used to detect if an obstacle is in front of the robot. $sc_{left}$ and $sc_{right}$ are the angular speeds calculated in the controller, which have to be sent to the robot over the radio channel; the range of these speeds go from 0%–100%.

The control law in Equation (4) modifies the wheel speed of the robot to force it to stay at a constant distance (50% of the lateral sensor’s range) from the wall with a constant speed (50% of the robot’s maximum speed).

This navigation algorithm is the same for the discrete-time solution and for the event-based one. In the mobile robot, when the discrete-time solution is used, every sampling time $1/f_{s}$, the robot sends the sensor information ($obt_{front\_left}$, $obt_{front\_right}$, $obt_{side\_left}$ and $obt_{side\_right}$) over the radio interface. After the controller receives this information, it computes the control signals ($sc_{left}$ and $sc_{right}$), and they are sent to the robot over the same radio interface. Finally, in the robot, the received control signals are assigned by the drive system to the wheels ($speed_{left}=sc_{left}$ and $speed_{right}=sc_{right}$).

**Figure 6:**
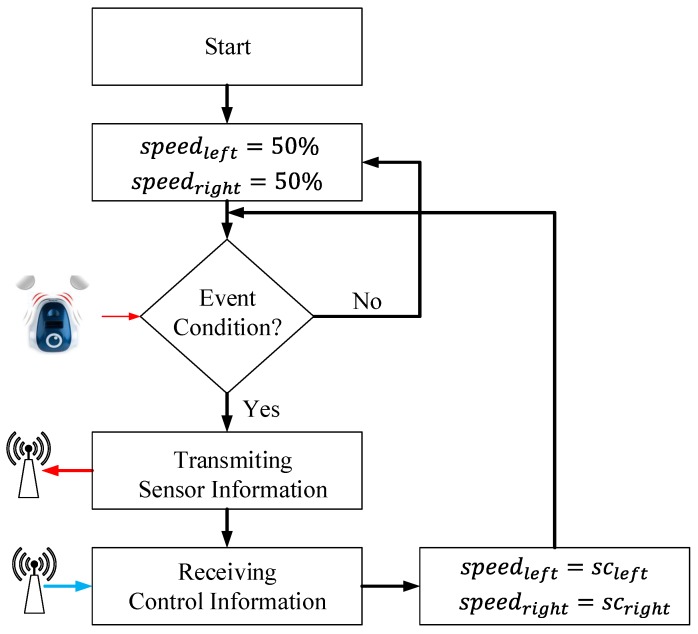
Event-based architecture for the mobile robots.

When the event-based proposal is considered, as was mentioned before, the algorithm in the controller is the same, but in the mobile robot, an event-based algorithm is implemented. The proposed event-based system for the mobile robot is depicted in Figure 6. The event-based algorithm in the robot depends on the event condition, which is presented in Equation (5), where *w* represents the reference signal, which is 50% for this algorithm. The event threshold $\bar{e}$ is the parameter that defines the accuracy of the system, the number of the events and its influence on the communication load in the RF interface.

(5) $$\begin{array}{c} \mathrm{if}\;(\left(obt_{front\_left}>0\right)\;\mathrm{OR}\;\left(abs\left(obt_{lat\_left}-w\right)>\bar{e}\right)\\ \left\{event=true\right\}\\ \mathrm{else}\;\{event=false\} \end{array}$$

When the event condition is satisfied, an event is generated, and the robot sends to the controller the sensor information. At the same time, the controller, after computing the control signals, returns to the robot this information, which contains new speed values. Every time the event condition is true, the RF channel is busy with the information that the robot and the controller are interchanging; the rest of the time, the communication channel is free.

### 5.2. Obstacle Avoidance Algorithm

**Figure 7:**
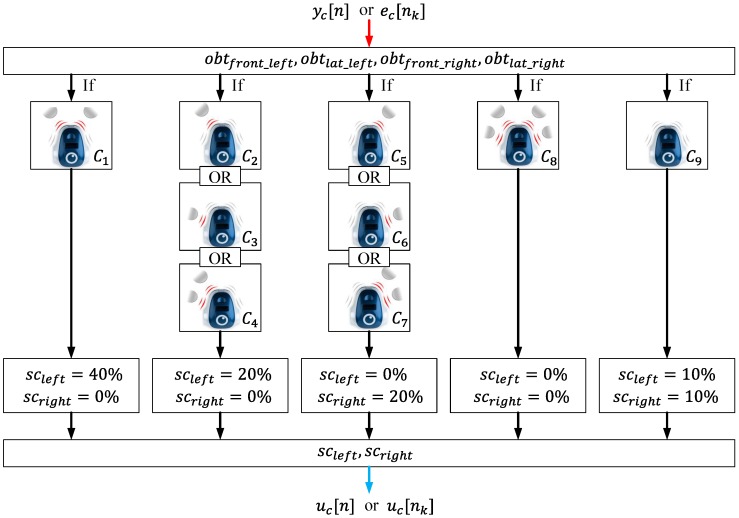
Block diagram of the obstacle avoidance algorithm.

An obstacle avoidance algorithm makes the robot able to reach a destination without any collisions. To analyze the event-based architectures proposed in this work, a simple obstacle avoidance algorithm has been proposed. It is depicted in Figure 7, and the control law is given by the Equation (6).

(6) $$\begin{array}{c} \mathrm{if}\;\left(C_{1}\right)\;\left\{sc_{left}=40\%,sc_{right}=0\%\right\}\\ \text{else if }\left(C_{2}\right)\;\mathrm{OR}\;\left(C_{3}\right)\;\mathrm{OR}\;\left(C_{4}\right)\;\left\{sc_{left}=20\%,sc_{right}=0\%\right\}\\ \text{else if }\left(C_{5}\right)\;\mathrm{OR}\;\left(C_{6}\right)\;\mathrm{OR}\;\left(C_{7}\right)\;\left\{sc_{left}=0\%,sc_{right}=20\%\right\}\\ \text{else if }\left(C_{8}\right)\;\left\{sc_{left}=0\%,sc_{right}=0\%\right\}\\ \text{else }\left(C_{9}\right)\;\left\{sc_{left}=10\%,sc_{right}=10\%\right\} \end{array}$$

where the conditions $C_{1}$–$C_{9}$ are defined by Equations (7)–(15).

(7) $$\text{if }\left(\left(obt_{front\_left}>0\right)\;\mathrm{AND}\;\left(obt_{front\_right}>0\right)\right)\;\left\{C_{1}=true\right\}$$

(8) $$\text{if }\left(obt_{front\_left}>0\right)\;\left\{C_{2}=true\right\}$$

(9) $$\text{if }\left(obt_{lat\_left}>0\right)\;\left\{C_{3}=true\right\}$$

(10) $$\text{if }\left(\left(obt_{front\_left}>0\right)\;\mathrm{AND}\;\left(obt_{lat\_left}>0\right)\right)\;\left\{C_{4}=true\right\}$$

(11) $$\text{if }\left(obt_{front\_right}>0\right)\;\left\{C_{5}=true\right\}$$

(12) $$\text{if }\left(obt_{lat\_right}>0\right)\;\left\{C_{6}=true\right\}$$

(13) $$\text{if }\left(\left(obt_{front\_right}>0\right)\;\mathrm{AND}\;\left(obt_{lat\_right}>0\right)\right)\;\left\{C_{7}=true\right\}$$

(14) $$\begin{array}{c} \text{if }(\left(obt_{front\_left}>0\right)\;\mathrm{AND}\;\left(obt_{lat\_left}>0\right)\;\mathrm{AND}\;\left(obt_{front\_right}>0\right)\;\mathrm{AND}\;\left(obt_{lat\_right}>0\right))\\ \left\{C_{8}=true\right\} \end{array}$$

(15) $$\begin{array}{c} \text{if }(\left(obt_{front\_left}=0\right)\;\mathrm{AND}\;\left(obt_{lat\_left}=0\right)\;\mathrm{AND}\;\left(obt_{front\_right}=0\right)\;\mathrm{AND}\;\left(obt_{lat\_right}=0\right))\\ \left\{C_{9}=true\right\} \end{array}$$

This obstacle avoidance algorithm calculates the speed of each wheel as a function of the information in the obstacle detection sensors. Then, this information is sent to the robot via an RF interface. In this case, as in the case of the wall following algorithm, the control law is the same for the discrete-time implementation and for the event-based control strategy proposed in this work.

In the discrete-time implementation, every sampling period ($1/f_{s}$), the robot sends to the controller the sensor information. At the same time, the controller sends to the robot the control signals, which contain the angular speeds to modify the trajectory of the robot.

For the event-based solution, the structure of the algorithm in the robot is the same as the one defined for the wall following algorithm depicted in Figure 6, with the following differences:

(1) the steady speeds are 10% for each wheel
(2) and the event condition is defined by Equation (16):
  (16) $$\begin{array}{c} \mathrm{if}\;(\left(obt_{front\_left}-w>\bar{e}\right)\;\mathrm{OR}\;\left(obt_{lat\_left}-w>\bar{e}\right)\;\mathrm{OR}\ldots \\ \ldots \left(obt_{front\_right}-w>\bar{e}\right)\;\mathrm{OR}\;\left(obt_{lat\_right}-w>\bar{e}\right))\\ \left\{event=true\right\}\\ \mathrm{else}\;\{event=false\} \end{array}$$

In this case, the *w* is the reference signal, which for the obstacle avoidance algorithm is 0%, and $\bar{e}$ is the event threshold of the system.

## 6. Experimental Results

**Figure 8:**
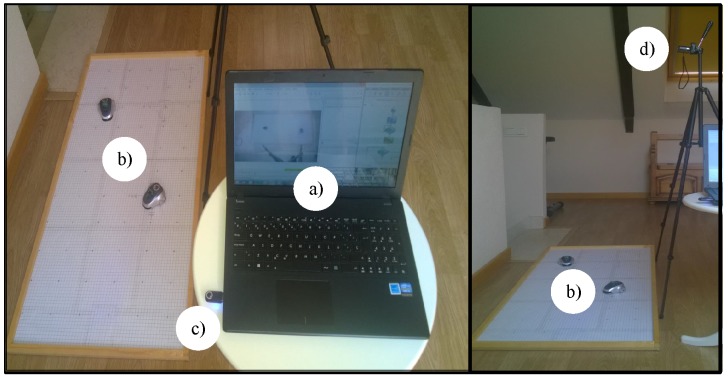
Components of the test laboratory: (**a**) controller; (**b**) mOway mobile robots; (**c**) RF interface; and (**d**) video camera.

To check the proposed event-based control strategy in this work, a test laboratory with the low cost mOway differential wheel drive robots has been developed; see Figure 8.

**Figure 9:**
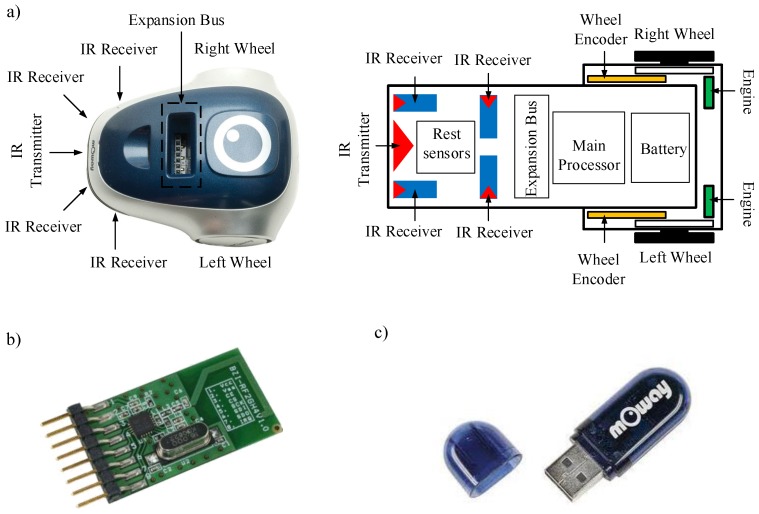
mOway mobile robot platform: (**a**) structure and components diagram; (**b**) RF module; and (**c**) RF USB interface.

The differential wheeled robot used in the experiments is depicted in Figure 9. The robots have four infra-red obstacle detection sensors, two in the front and the other two on the sides with a maximum range of 3cm. The drive system of the robots permits angular speeds from 0 rad/s–10.9 rad/s. The dimensions regarding the dynamic model are: the distance between the wheel L=6.6cm and the wheel’s radio R=1.6cm.

The controller has been implemented in a laptop and the navigation algorithm in C++ with the Windows OS. In the robots, the discrete-time and the event-based solutions were programmed in the mOway World application. In both cases, the discrete-time and the event-based structures, the robot works with a sampling frequency of $f_{s}=10$ Hz. A radio frequency link of 2.4GHz has been used for communication between the robot and the controller. Finally, a video camera and the Tracker video processing tool were utilized to capture the real path of the robots.

The wireless communication systems (RF module and RF USB) are based on the nRF24L01 transceiver manufactured by Nordic Semiconductors. The RF interface works in the worldwide ISM frequency band at 2.400–2.4835 GHz and uses GFSK modulation. It has user-configurable parameters, like frequency channel, output power and air data rate. The air data rate for each channel is configurable to 2 Mbps, and 126 channels can be configured. The used bandwidth in the system depends on the traffic in the wireless network. The control system proposed in this work uses less control information than the classical discrete time solution. In this case, the bandwidth not utilized to send control information can be used to transmit other user information or to include more agents in the system.

The wall following and the obstacle avoidance navigation algorithms, which were defined in Section 5, have been checked in the test laboratory using the two control strategies, the proposed event-based control and the classical discrete-time control. The obtained results and a comparison between them are presented in the next sections.

### 6.1. Wall Following

**Figure 10:**
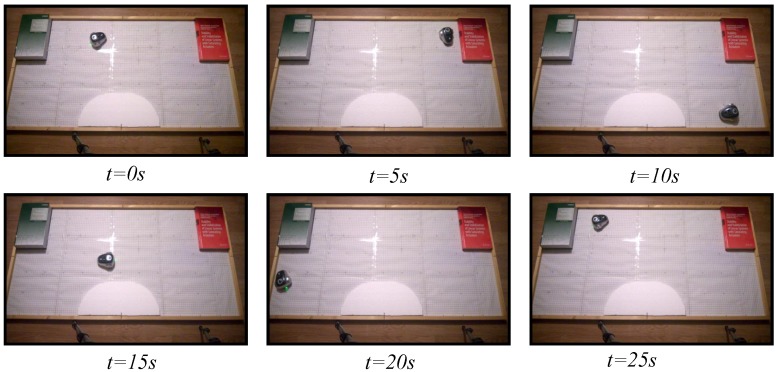
Experiment 1. Wall following algorithm using discrete-time architecture.

**Figure 11:**
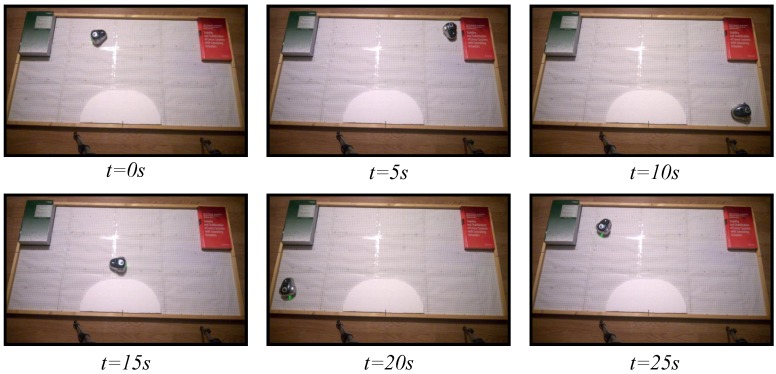
Experiment 2. Wall following algorithm using event-based architecture with $\bar{e}=10\%$.

**Figure 12:**
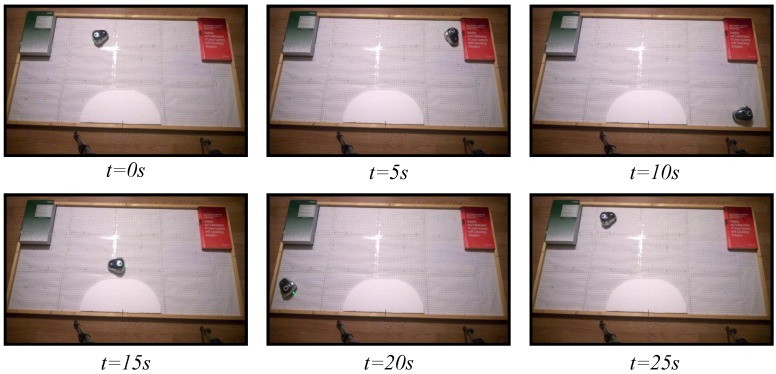
Experiment 3. Wall following algorithm using event-based architecture with $\bar{e}=15\%$.

The wall following algorithm described in Section 5.1 has been programmed in the test laboratory using the discrete-time architecture and the proposed event-based strategy. To check the response of both strategies, three experiments have been set: one with the discrete-time architecture and two with the event-based strategy. For the event-based solution, two event thresholds have been set, an event threshold of $\bar{e}=10\%$ for the second experiment and $\bar{e}=15\%$ for the third one. In all experiments, the robot has been navigating during 30 s in an environment with different objects as in Figure 10, Figure 11 and Figure 12 show.

**Figure 13:**
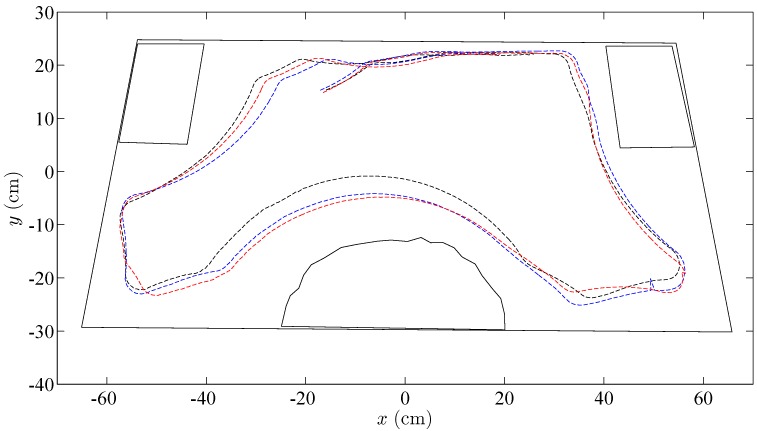
Trajectories of the robots controlled by the wall following algorithm. The black dashed line is the discrete-time strategy; the blue dashed line represents the event-based strategy with $\bar{e}=10\%$; and the red dashed line is the event-based strategy with $\bar{e}=15\%$.

In the three cases, the video of the experiments has been analyzed with the tracker tool, and the trajectories of the robots have been obtained. As shown in Figure 13, the three algorithms resolve the navigation problem in the correct way, and the trajectories of the robots are very similar.

**Figure 14:**
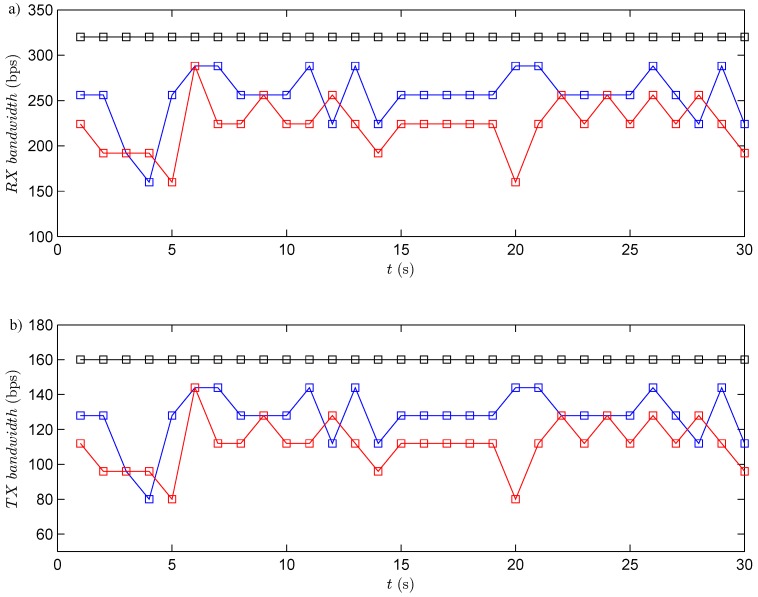
RF channel utilization. The black square is the discrete-time strategy; the blue square represents the event-based strategy with $\bar{e}=10\%$; and and red dashed line is the event-based strategy with $\bar{e}=15\%$. (**a**) Bandwidth used in reception (from the robots to the controller); (**b**) bandwidth used in transmission (from the controller to the robots).

The RF channel utilization has been analyzed for the three experiments. In this case, in each period, the robot transmits the information of its four obstacles sensors (the information of each sensor is coded by eight bits, in total 32 bits per each transmission). At the same time, the controller sends to the robot the speed of each wheel (the angular speed of each wheel is coded by eight bits, this means 16 bits per each transmission). Taking into account the previous considerations, the bandwidth utilized by the system is presented in Figure 14. The results show that the event-based solutions have a higher efficiency *versus* the discrete-time one. In the event-based case, the channel utilization also depends on the selected event threshold. If the event threshold increases, the efficiency in the RF channel increases, as well. The event threshold has to be selected carefully, because of if this parameter is too high, the channel utilization decreases, but the navigation problem cannot be resolved with the required accuracy.

To study the efficiency of the proposed solution, the number of accumulated transmissions of these experiments has been calculated. In Table 1, this parameter is presented for the three experiments.

**Table 1:** RF channel utilization in the wall following algorithm. Number of accumulated transmissions.

t (s)	Discrete-time	Event-based $\left(\bar{e}=10\%\right)$	Event-based $\left(\bar{e}=15\%\right)$
5	50	35	31
10	100	77	69
15	150	117	104
20	200	158	137
25	250	199	174
30	300	239	210

The results of Table 1 clearly show that the proposed event-based solution needs to use the RF channel less than a classical discrete-time one. The presented architecture has about 20.3% efficiency with $\bar{e}=10\%$ and about 30% efficiency with $\bar{e}=15\%$.

### 6.2. Obstacle Avoidance

To study the performance of the proposed architecture in an environment of multiple robots, the obstacle avoidance algorithm presented in Section 5.2 has been implemented in the test laboratory. In this case, two mOway robots have been used, and the two strategies, the discrete-time and the event-based, have been analyzed. Two experiments have been set up. In the fist one, the classical discrete-time solution has been used. In the second experiment, the proposed event-based solution has been programmed with an event threshold $\bar{e}=0\%$ to check the worst case in the event-based solution. As in the experiments of the wall following algorithms, in this case, the robots have been controlled by the navigation algorithm for 30 s.

**Figure 15:**
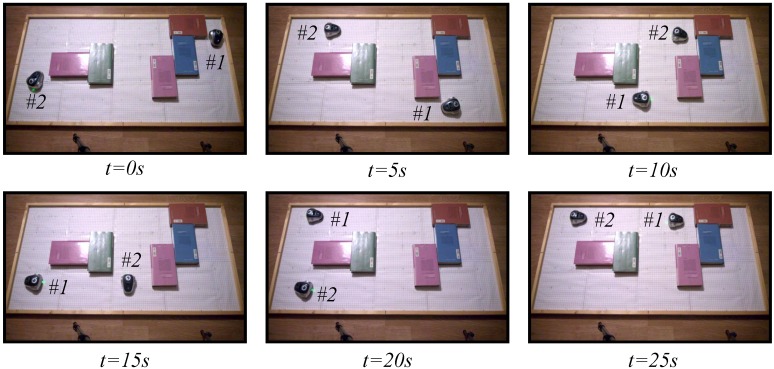
Experiment 1. The obstacle avoidance algorithm using a discrete-time strategy with a centralized controller and two robots. The symbols #1 and #2 represent Robot 1 and Robot 2, respectively.

**Figure 16:**
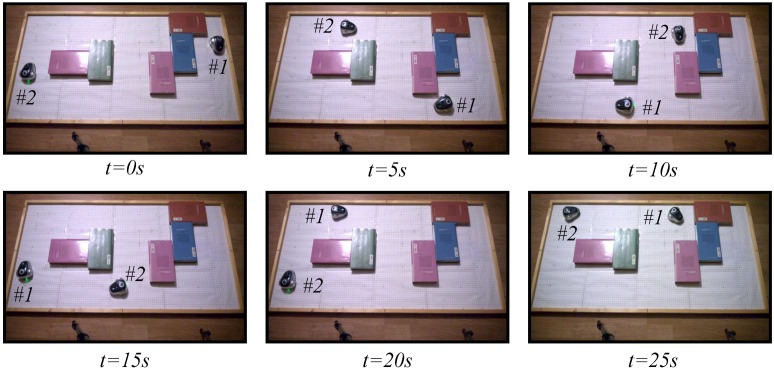
Experiment 2. The obstacle avoidance algorithm using an event-based strategy with a centralized controller and two robots. The symbols #1 and #2 represent Robot 1 and Robot 2, respectively.

**Figure 17:**
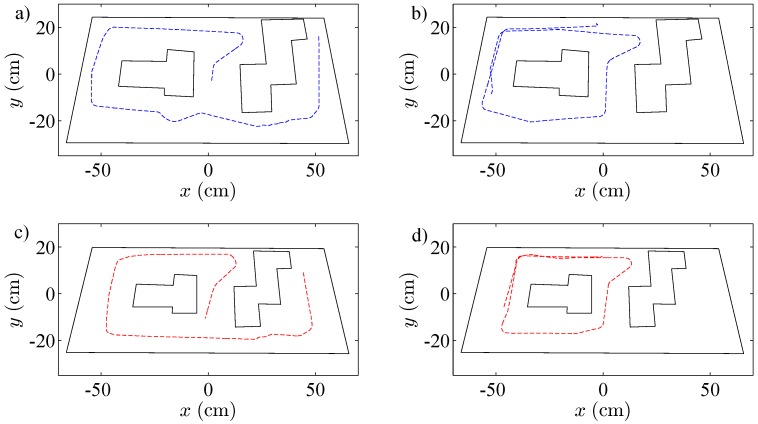
Trajectories of the robots controlled by the obstacle avoidance algorithm. The blue dashed line represents the discrete-time solution. (**a**) Robot 1; (**b**) Robot 2. The red dashed line is the event-based strategy. (**c**) Robot 1; (**d**) Robot 2.

The snapshots of these two experiments are depicted in Figure 15 and Figure 16. The videos of the experiments were analyzed by the tracker tool to obtain the trajectories of the different robots; they are presented in Figure 17. In the two experiments, the navigation problem was resolved in a satisfactory way and without instabilities in the system.

**Figure 18:**
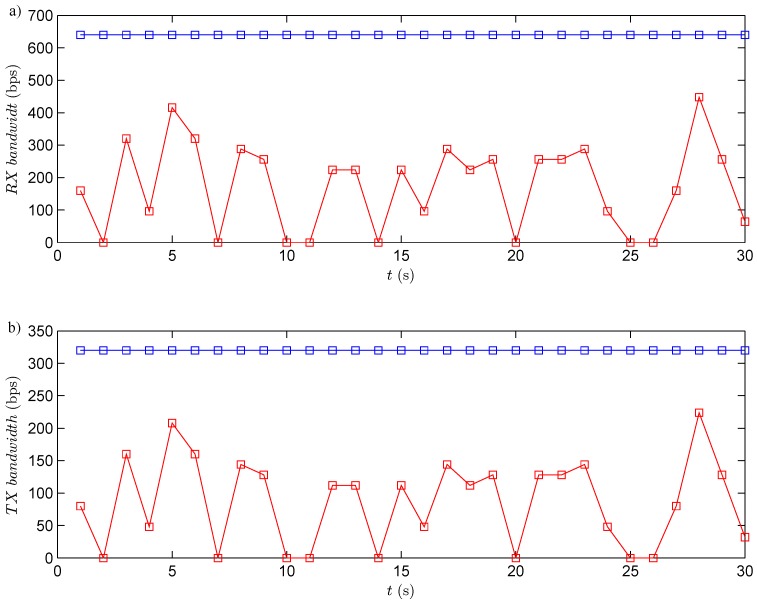
RF channel utilization. The blue square shows the discrete-time strategy; the red square represents the event-based strategy. (**a**) Bandwidth used in reception (from the robots to the controller); (**b**) bandwidth used in transmission (from the controller to the robots).

As in the previous experiment, every period, the robots send to the controller the information of their obstacle sensors (each robot sends 32 bits), and the controller transmits to the robots the angular speeds (16 bits per each robot). The used bandwidth in both directions is presented in Figure 18. The results show that the event-based solution has a higher efficiency in communication resource utilization than the discrete-time one.

In Table 2, the number of accumulated transmissions of these experimented is presented.

The results in Table 2 show that the presented event-based control strategy has a higher efficiency in the communication resource usage than the discrete-time solution. In the analyzed experiments, the proposed strategy obtains an improvement of 73%.

**Table 2:** RF channel utilization. Number of accumulated transmissions.

t (s)	Discrete-Time	Event-Based
5	100	31
10	200	58
15	300	79
20	400	106
25	500	134
30	600	163

## 7. Conclusions and Future Work

The proposed event-based control strategy can be implemented in mobile robot architectures in a simple way. In this work, these strategies are applied to the mOway mobile robots platform, but this solution can be adapted to other kinds of robots following the same methodology. The control structures defined in the mobile robot only need to set one parameter, the event threshold $\bar{e}$, which defines the accuracy of the system and the performance in the radio interface. In the navigation experiments presented in this work, the proposed system has resolved the navigation problem in the same way as the discrete-time solution. The event-based solution obtains higher efficiencies in communication resource usage than the classical discrete-time solution. In the wall following algorithm, an efficiency of 15% is obtained, and for the obstacle avoidance algorithm, it is 73%.

As future work, the system is now being checked with a hybrid system, which contains several navigation algorithms (go to goal, wall following in its two versions and obstacle avoidance) working together using the architectures presented in this paper. Furthermore, the stability of the proposed solution is going to be analyzed in a theoretical way.
